# Smart campus: Data on energy consumption in an ICT-driven university

**DOI:** 10.1016/j.dib.2017.11.091

**Published:** 2017-12-07

**Authors:** Segun I. Popoola, Aderemi A. Atayero, Theresa T. Okanlawon, Benson I. Omopariola, Olusegun A. Takpor

**Affiliations:** a Department of Electrical and Information Engineering, Covenant University, Ota, Nigeria; b Physical Planning and Development Unit, Covenant University, Ota, Nigeria

**Keywords:** Smart campus, Energy consumption, Energy efficiency, Load forecasting, Energy management

## Abstract

In this data article, we present a comprehensive dataset on electrical energy consumption in a university that is practically driven by Information and Communication Technologies (ICTs). The total amount of electricity consumed at Covenant University, Ota, Nigeria was measured, monitored, and recorded on daily basis for a period of 12 consecutive months (January–December, 2016). Energy readings were observed from the digital energy meter (EDMI Mk10E) located at the distribution substation that supplies electricity to the university community. The complete energy data are clearly presented in tables and graphs for relevant utility and potential reuse. Also, descriptive first-order statistical analyses of the energy data are provided in this data article. For each month, the histogram distribution and time series plot of the monthly energy consumption data are analyzed to show insightful trends of energy consumption in the university. Furthermore, data on the significant differences in the means of daily energy consumption are made available as obtained from one-way Analysis of Variance (ANOVA) and multiple comparison post-hoc tests. The information provided in this data article will foster research development in the areas of energy efficiency, planning, policy formulation, and management towards the realization of smart campuses.

**Specifications Table**

**Table:** 

Subject area	*Engineering*
More specific subject area	*Electrical/Power Engineering*
Type of data	*Tables, graphs, figures, and spreadsheet file*
How data was acquired	*Daily energy data were obtained from the Liquid Crystal Display (LCD) of the Digital Energy Meter (EDMI Mk10E) located at the distribution substation that supplies electricity to Covenant University, Ota, Nigeria.*
Data format	*Raw, analyzed*
Experimental factors	*Data monitoring and logging were performed manually i.e. the recording process was not automated*
Experimental features	*Statistical analyses of the monthly data were performed to show the trends of energy consumption in an ICT-driven university community*
Data source location	*The energy data provided in this article were collected at Covenant University, Canaanland, Ota, Nigeria (Latitude 6.6718*°*N, Longitude 3.1581°E)*
Data accessibility	*A comprehensive energy consumption dataset is provided in this article*

**Value of the data**

• Free accessibility to energy consumption data of an ICT-driven university will encourage more evidence-based (empirical) research for better understanding of electricity consumption pattern and improvement in energy consumption efficiency [1], [2], [3].
• Researchers, engineers, and industry experts will find the data provided in this article useful for energy consumption model development, energy audit, load forecasting, and energy management [4], [5], [6].
• Statistical analyses of the electrical load demands will assist energy policy makers and university management in proper energy audit, planning, budgeting, and decision-making [7].
• Public availability of these energy data is considered valuable to the timely actualization of smart campuses as it relates to sustainable development [8], [9], [10].

## 1 Data

ICTs enable global interconnectedness that is required for the delivery of quality education [11]. However, ICTs require functional supplies of electrical energy to operate. As a matter of fact, universities of the 21st century are practically driven by ICTs [11]. Therefore, the electrical load demands of facilities and services within the university community must be satisfactorily met to guarantee sustainable education. The data that are made publicly available in this article contain useful information about the electrical energy consumption in an ICT-driven university community. The total amount of electricity consumed at Covenant University, Ota, Nigeria was measured, monitored, and recorded on daily basis for a period of 12 consecutive months (January–December, 2016).

**Fig. 1:**
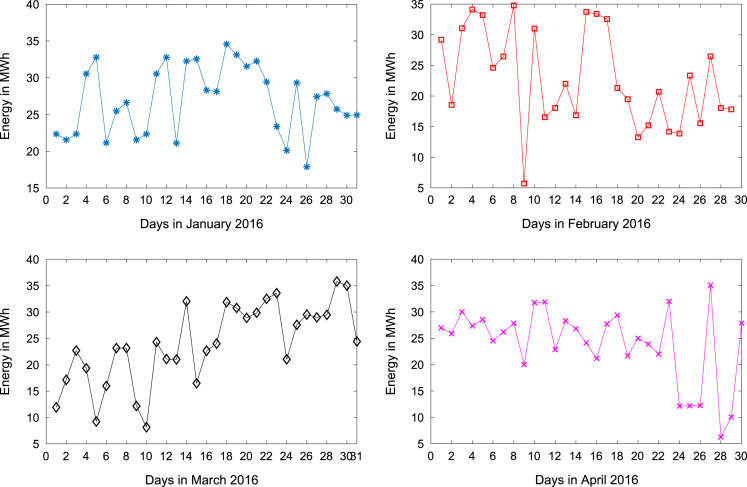
Trends of energy consumption in January–April 2016.

**Fig. 2:**
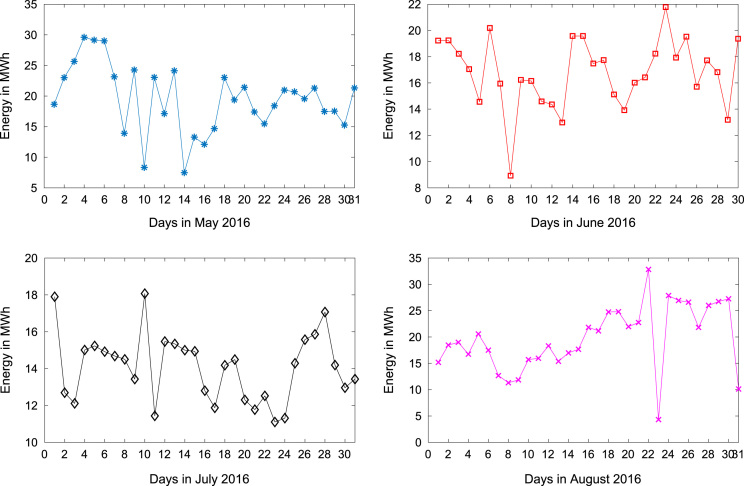
Trends of energy consumption in May–August 2016.

**Fig. 3:**
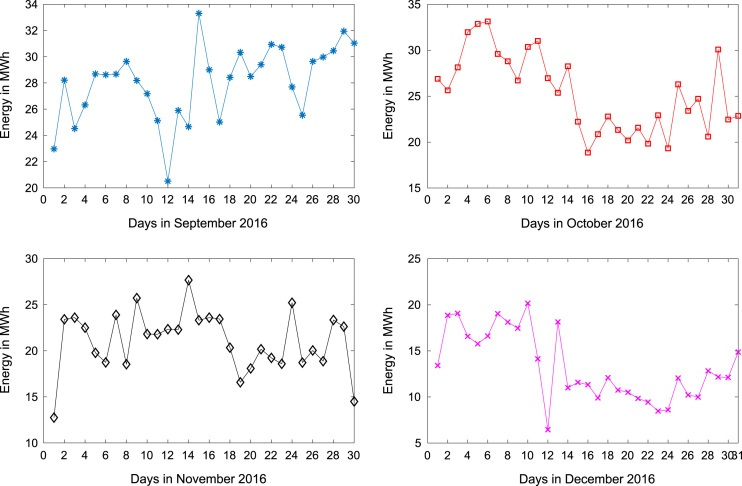
Trends of energy consumption in September–December 2016.

**Fig. 4:**
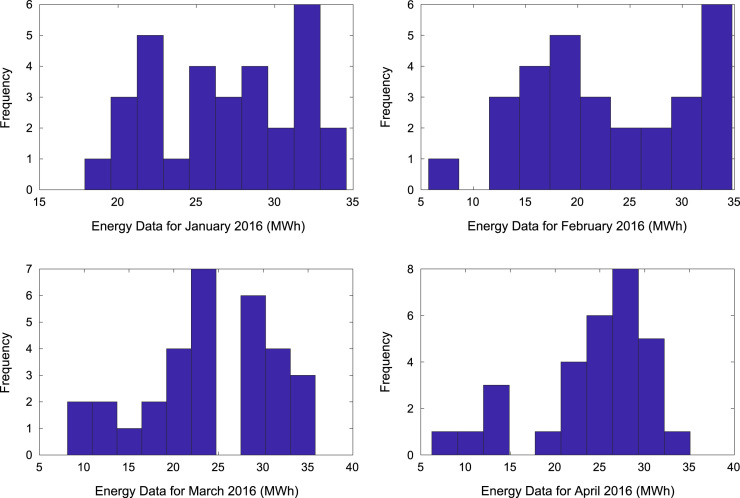
Histogram plot of energy consumption in January–April 2016.

**Fig. 5:**
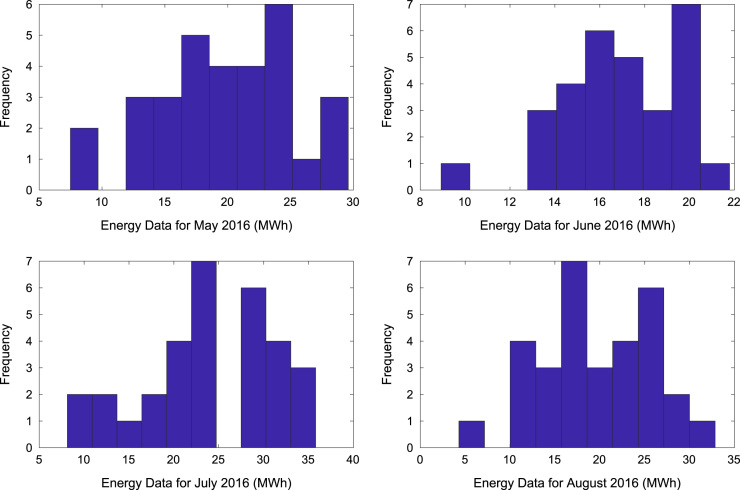
Histogram plots of energy consumption in May–August 2016.

**Fig. 6:**
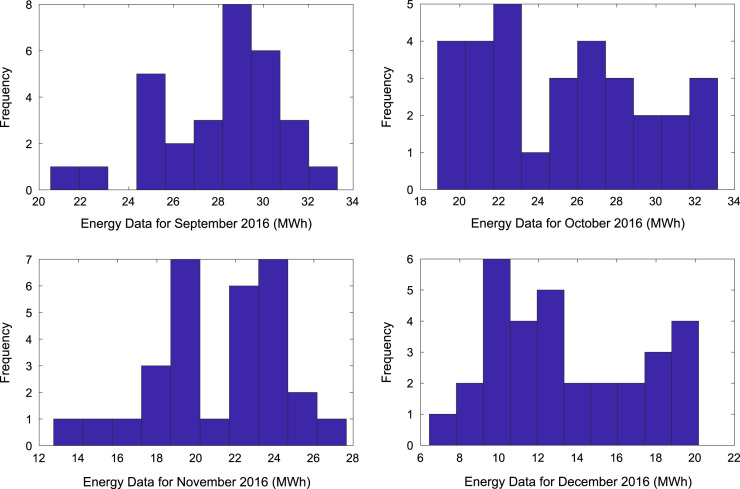
Histogram plot of energy consumption in September–December 2016.

Table 1 presents the daily energy consumption readings at Covenant University from January to December 2016. These data can be explored to gain useful insights about the load demands of the university community across all weather seasons. In addition, descriptive first-order statistics are presented in Table 2 to explain the data distribution of the electricity consumption. Fig. 1, Fig. 2, Fig. 3 show the trends of energy consumption for each month in 2016. The graphs were plotted using MATLAB 2017b computational software. Histogram plots of the monthly energy data are illustrated in Fig. 4, Fig. 5, Fig. 6 to show the statistical distribution of the data. Proper interpretations and discussions of these plots will give useful insights that are needed for valid conclusions.

**Table 1:** Daily energy consumption readings at Covenant University for the Year 2016.

Day	Daily energy consumption (MWh)											
	*Jan*	*Feb*	*Mar*	*Apr*	*May*	*Jun*	*Jul*	*Aug*	*Sep*	*Oct*	*Nov*	*Dec*
1	22.35	29.18	11.91	26.97	18.64	19.23	17.9	15.19	22.97	26.89	12.74	13.41
2	21.55	18.55	17.12	25.87	23	19.24	12.69	18.46	28.21	25.64	23.41	18.86
3	22.35	31.04	22.7	30.03	25.65	18.22	12.11	18.99	24.52	28.14	23.59	19.07
4	30.525	34.11	19.33	27.36	29.59	17.05	15.01	16.74	26.32	31.97	22.5	16.58
5	32.78	33.21	9.21	28.55	29.14	14.56	15.23	20.58	28.69	32.87	19.77	15.77
6	21.15	24.6	15.97	24.5	29.01	20.19	14.92	17.5	28.62	33.14	18.73	16.61
7	25.475	26.44	23.16	26.17	23.14	15.95	14.68	12.66	28.67	29.6	23.89	19.04
8	26.625	34.77	23.18	27.84	13.91	8.93	14.5	11.3	29.64	28.8	18.53	18.12
9	21.55	5.72	12.16	20.03	24.26	16.23	13.43	11.85	28.18	26.71	25.71	17.46
10	22.35	31	8.13	31.77	8.34	16.15	18.07	15.71	27.18	30.37	21.81	20.17
11	30.525	16.53	24.3	31.89	23.05	14.59	11.43	15.96	25.12	31.01	21.79	14.13
12	32.78	18.04	21.08	22.85	17.12	14.36	15.47	18.31	20.51	26.97	22.34	6.45
13	21.1	22	21	28.31	24.13	12.98	15.34	15.38	25.9	25.37	22.29	18.14
14	32.26	16.85	32.03	26.79	7.48	19.58	15	16.98	24.66	28.26	27.66	11
15	32.56	33.7	16.49	24.12	13.29	19.58	14.94	17.67	33.3	22.22	23.31	11.58
16	28.3	33.4	22.64	21.16	12.11	17.48	12.8	21.83	29	18.86	23.59	11.33
17	28.12	32.55	24.01	27.72	14.67	17.74	11.87	21.17	25.02	20.86	23.44	9.9
18	34.57	21.31	31.88	29.37	23.02	15.12	14.18	24.75	28.42	22.79	20.33	12.09
19	33.11	19.47	30.78	21.67	19.37	13.92	14.49	24.8	30.33	21.32	16.57	10.75
20	31.55	13.27	28.89	24.98	21.42	16.02	12.3	21.97	28.49	20.18	18.08	10.51
21	32.27	15.2	29.87	23.88	17.39	16.42	11.77	22.75	29.39	21.57	20.17	9.84
22	29.42	20.69	32.51	21.99	15.46	18.23	12.52	32.84	30.94	19.83	19.22	9.44
23	23.39	14.18	33.59	32	18.4	21.78	11.1	4.31	30.72	22.92	18.58	8.47
24	20.11	13.87	21.03	12.14	20.97	17.93	11.31	27.87	27.69	19.32	25.21	8.6
25	29.31	23.32	27.58	12.18	20.69	19.53	14.29	26.93	25.54	26.29	18.71	12.05
26	17.88	15.55	29.5	12.22	19.55	15.71	15.57	26.61	29.64	23.41	20.03	10.22
27	27.43	26.47	28.99	35.08	21.3	17.72	15.86	21.82	29.97	24.72	18.86	9.99
28	27.83	18.04	29.47	6.23	17.47	16.82	17.07	26	30.45	20.6	23.34	12.83
29	25.72	17.81	35.79	10.04	17.53	13.19	14.19	26.74	31.94	30.08	22.62	12.17
30	24.89	N/A	35.02	27.85	15.25	19.37	12.96	27.25	31.03	22.46	14.49	12.12
31	24.93	N/A	24.42	N/A	21.32	N/A	13.43	10.12	N/A	22.86	N/A	14.87

**Table 2:** Descriptive first-order statistics of the 2016 energy consumption data.

Parameter	Monthly energy consumption (MWh)											
	*Jan*	*Feb*	*Mar*	*Apr*	*May*	*Jun*	*Jul*	*Aug*	*Sep*	*Oct*	*Nov*	*Dec*
Mean	27.07	22.79	23.60	23.92	19.62	16.71	14.14	19.78	27.93	25.54	21.27	13.26
Median	27.83	21.31	23.18	25.87	19.55	16.82	14.49	18.99	28.49	25.64	21.81	12.09
Standard Deviation	4.82	7.88	7.57	7.18	5.61	2.66	1.90	6.04	2.81	4.35	3.08	3.87
Variance	23.19	62.15	57.35	51.57	31.46	7.08	3.60	36.54	7.89	18.89	9.51	14.96
Kurtosis	1.74	2.03	2.26	3.11	2.74	3.87	2.39	3.16	3.23	1.82	3.53	1.85
Skewness	−0.19	0.02	−0.41	−0.93	−0.22	−0.65	0.19	−0.20	−0.59	0.13	−0.45	0.31
Range	16.69	29.05	27.66	28.85	22.11	12.85	6.97	28.53	12.79	14.28	14.92	13.72
Minimum	17.88	5.72	8.13	6.23	7.48	8.93	11.10	4.31	20.51	18.86	12.74	6.45
Maximum	34.57	34.77	35.79	35.08	29.59	21.78	18.07	32.84	33.30	33.14	27.66	20.17
Sum	784.94	660.87	684.30	693.71	569.10	484.45	410.04	573.67	810.03	740.71	616.82	384.58

## 2 Experimental design, materials and methods

**Fig. 7:**
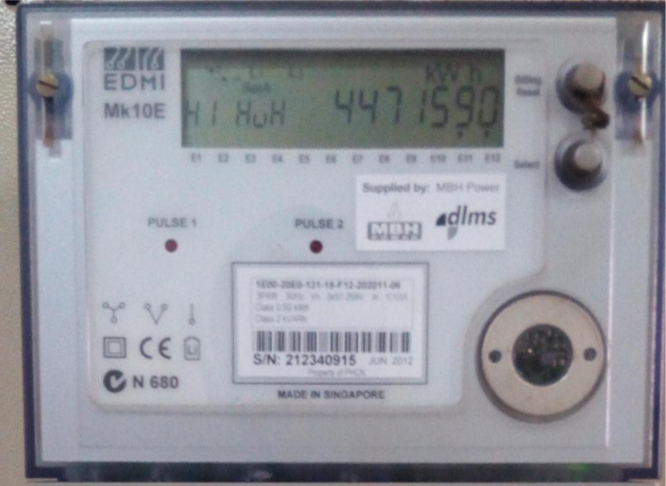
Electricity meter (EDMI Mk10E) Display.

**Fig. 8:**
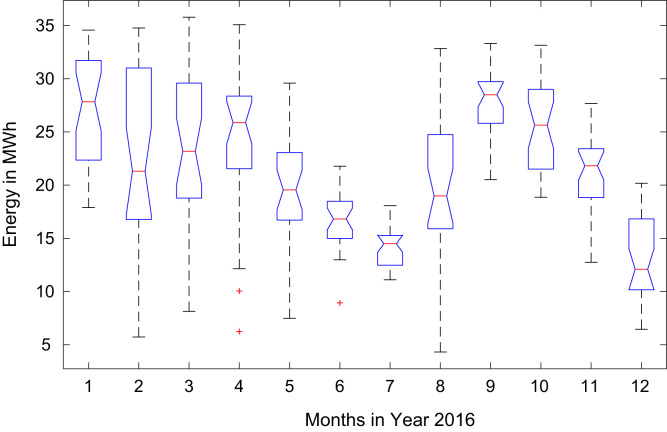
Box plot of energy consumption data.

**Fig. 9:**
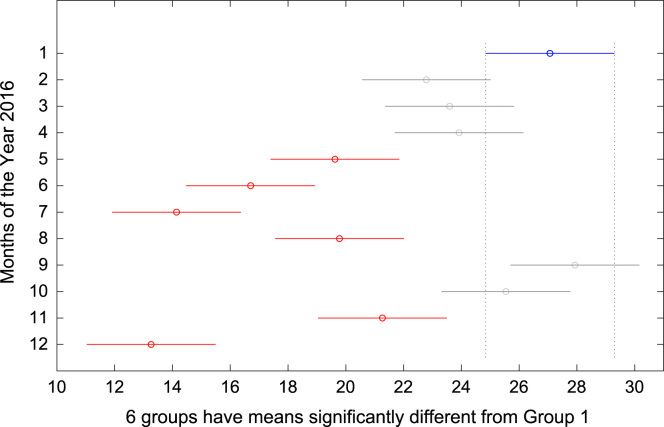
Post-Hoc test for January 2016.

**Fig. 10:**
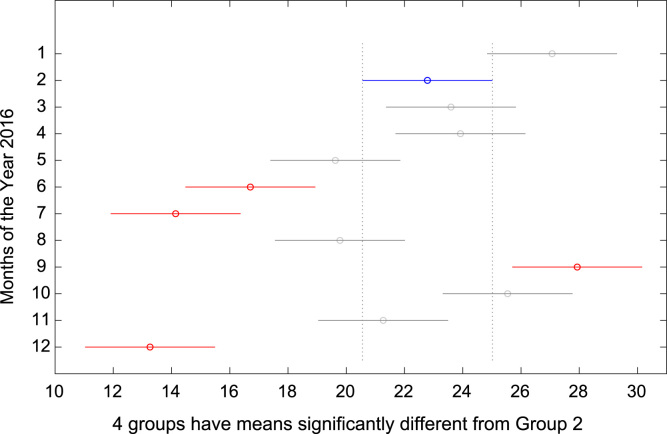
Post-Hoc test for February 2016.

**Fig. 11:**
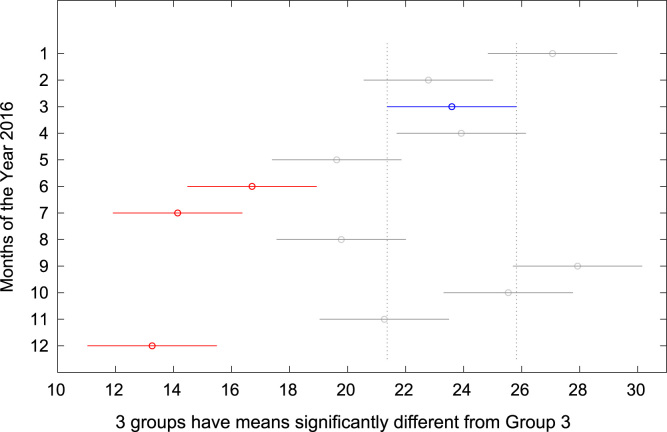
Post-Hoc test for March 2016.

**Fig. 12:**
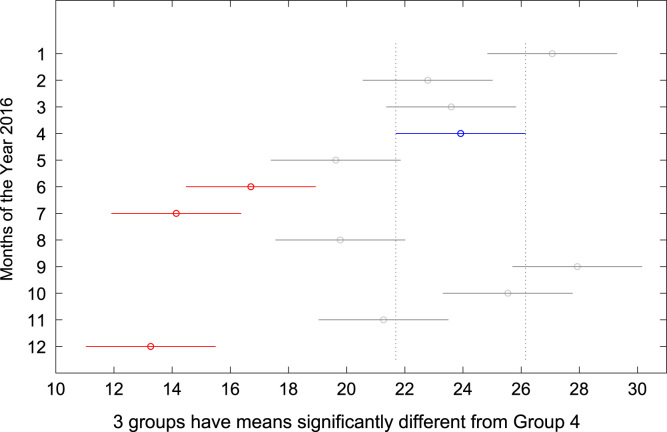
Post-Hoc test for April 2016.

**Fig. 13:**
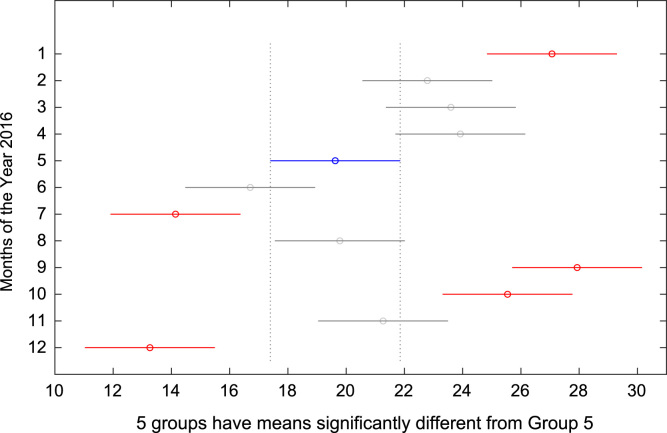
Post-Hoc test for May 2016.

**Fig. 14:**
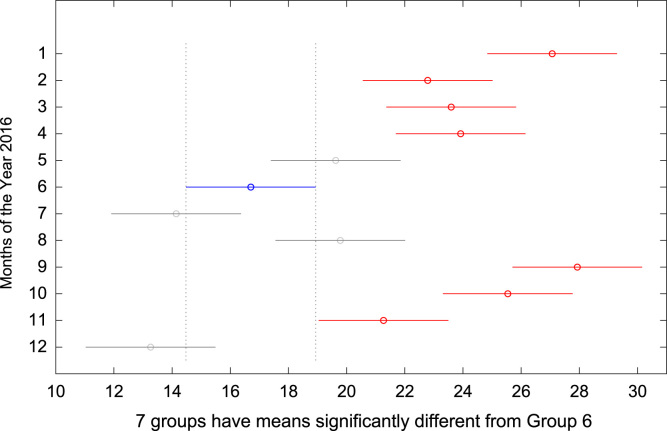
Post-Hoc test for June 2016.

**Fig. 15:**
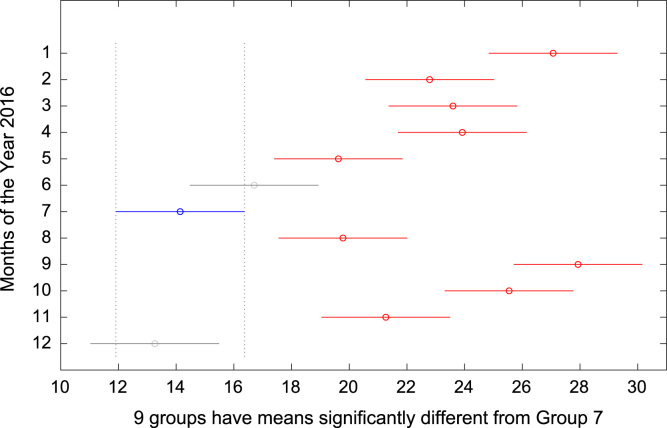
Post-Hoc test for July 2016.

**Fig. 16:**
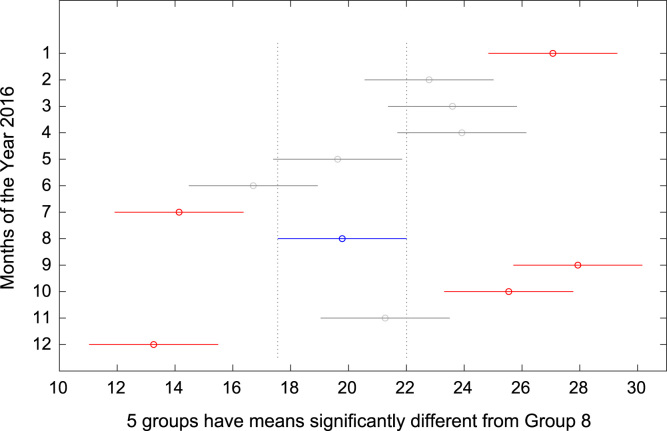
Post-Hoc test for August 2016.

**Fig. 17:**
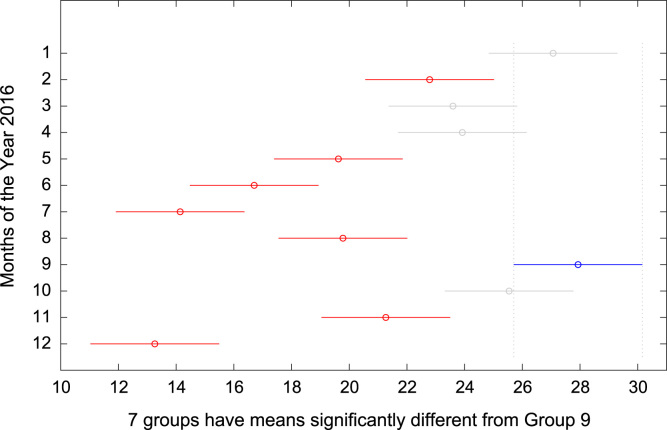
Post-Hoc test for September 2016.

**Fig. 18:**
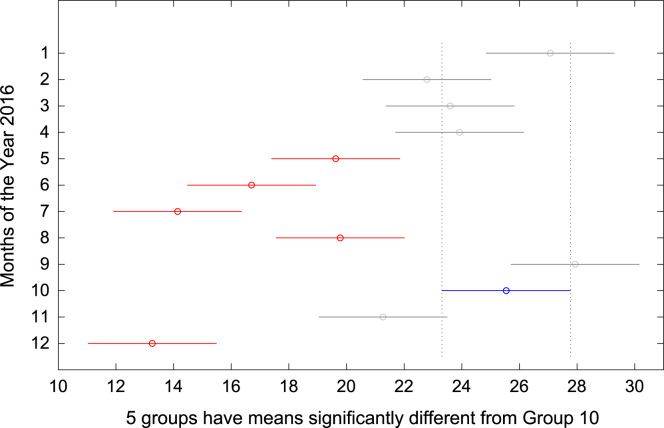
Post-Hoc test for October 2016.

**Fig. 19:**
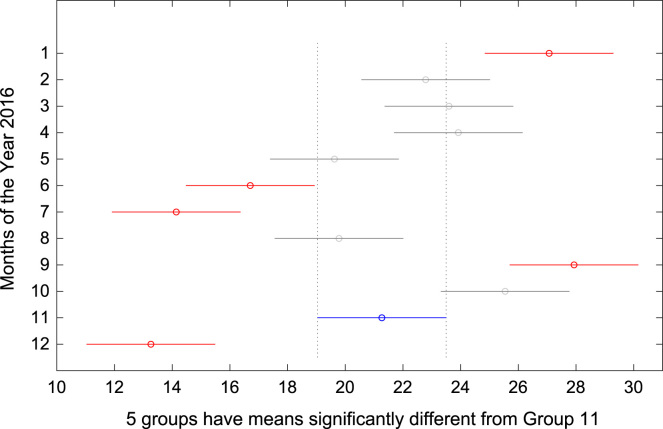
Post-Hoc test for November 2016.

**Fig. 20:**
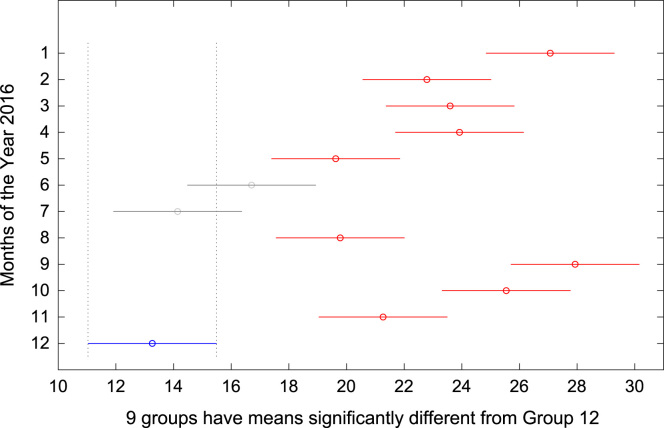
Post-Hoc test for December 2016.

The total amount of electricity consumed at Covenant University, Ota, Nigeria was measured, monitored, and recorded on daily basis for a period of 12 consecutive months (January–December, 2016). Covenant University is fully residential with modern hostel facilities and conducive accommodation for students and staff respectively. Detailed information about the electrical service areas is provided in [12]. Energy readings were observed from the digital energy meter (EDMI Mk10E) located at the distribution substation that supplies electricity to the university community. The energy display on the measuring instrument is shown in Fig. 7. The statistical analyses of the complete energy data are clearly presented for relevant utility and potential reuse. Data on the significant differences in the means of daily energy consumption are presented in Table 3. Monthly groups of energy data are depicted through their quartiles using box plot as shown in Fig. 8. Multiple comparison post-hoc tests were conducted to identify the groups with significant differences and their respective mean differences. The statistical data are presented in Table 4 and Fig. 9, Fig. 10, Fig. 11, Fig. 12, Fig. 13, Fig. 14, Fig. 15, Fig. 16, Fig. 17, Fig. 18, Fig. 19, Fig. 20.

**Table 3:** ANOVA test.

Source of variation	Sum of squares	Degree of freedom	Mean squares	F statistic	Prob > F
Columns	7299.75	11	663.614	24.56	5.094 × 10^–37^
Error	9077.26	336	27.016		
Total	16377.01	347

**Table 4:** Multiple comparison post-hoc test.

Groups Compared		Lower limits for 95% confidence intervals	Mean difference	Upper limits for 95% confidence intervals	*p*-value
Jan	Feb	−0.1825	4.2783	8.7390	0.0745
Jan	Mar	−0.9904	3.4703	7.9311	0.3133
Jan	Apr	−1.3149	3.1459	7.6066	0.4731
Jan	May	2.9820	7.4428	11.9035	0.0001
Jan	Jun	5.9010	10.3617	14.8225	0.0001
Jan	Jul	8.4669	12.9276	17.3883	0.0001
Jan	Aug	2.8244	7.2852	11.7459	0.0001
Jan	Sep	−5.3259	−0.8652	3.5956	1.0000
Jan	Oct	−2.9356	1.5252	5.9859	0.9940
Jan	Nov	1.3365	5.7972	10.2580	0.0013
Jan	Dec	9.3448	13.8055	18.2663	0.0001
Feb	Mar	−5.2687	−0.8079	3.6528	1.0000
Feb	Apr	−5.5931	−1.1324	3.3283	0.9996
Feb	May	−1.2963	3.1645	7.6252	0.4633
Feb	Jun	1.6227	6.0834	10.5442	0.0005
Feb	Jul	4.1886	8.6493	13.1100	0.0001
Feb	Aug	−1.4538	3.0069	7.4676	0.5474
Feb	Sep	−9.6042	−5.1434	−0.6827	0.0090
Feb	Oct	−7.2138	−2.7531	1.7076	0.6819
Feb	Nov	−2.9418	1.5190	5.9797	0.9942
Feb	Dec	5.0665	9.5272	13.9880	0.0001
Mar	Apr	−4.7852	−0.3245	4.1363	1.0000
Mar	May	−0.4883	3.9724	8.4331	0.1371
Mar	Jun	2.4306	6.8914	11.3521	0.0001
Mar	Jul	4.9965	9.4572	13.9180	0.0001
Mar	Aug	−0.6459	3.8148	8.2756	0.1820
Mar	Sep	−8.7963	−4.3355	0.1252	0.0659
Mar	Oct	−6.4059	−1.9452	2.5156	0.9589
Mar	Nov	−2.1338	2.3269	6.7876	0.8666
Mar	Dec	5.8744	10.3352	14.7959	0.0001
Apr	May	−0.1638	4.2969	8.7576	0.0716
Apr	Jun	2.7551	7.2159	11.6766	0.0001
Apr	Jul	5.3210	9.7817	14.2425	0.0001
Apr	Aug	−0.3214	4.1393	8.6000	0.0992
Apr	Sep	−8.4718	−4.0110	0.4497	0.1275
Apr	Oct	−6.0814	−1.6207	2.8400	0.9900
Apr	Nov	−1.8094	2.6514	7.1121	0.7323
Apr	Dec	6.1989	10.6597	15.1204	0.0001
May	Jun	−1.5418	2.9190	7.3797	0.5948
May	Jul	1.0241	5.4848	9.9456	0.0034
May	Aug	−4.6183	−0.1576	4.3031	1.0000
May	Sep	−12.7687	−8.3079	−3.8472	0.0001
May	Oct	−10.3783	−5.9176	−1.4569	0.0009
May	Nov	−6.1063	−1.6455	2.8152	0.9887
May	Dec	1.9020	6.3628	10.8235	0.0002
Jun	Jul	−1.8949	2.5659	7.0266	0.7721
Jun	Aug	−7.5373	−3.0766	1.3842	0.5100
Jun	Sep	−15.6876	−11.2269	−6.7662	0.0001
Jun	Oct	−13.2973	−8.8366	−4.3758	0.0001
Jun	Nov	−9.0252	−4.5645	−0.1037	0.0394
Jun	Dec	−1.0169	3.4438	7.9045	0.3253
Jul	Aug	−10.1031	−5.6424	−1.1817	0.0021
Jul	Sep	−18.2535	−13.7928	−9.3320	0.0001
Jul	Oct	−15.8631	−11.4024	−6.9417	0.0001
Jul	Nov	−11.5911	−7.1303	−2.6696	0.0001
Jul	Dec	−3.5828	0.8779	5.3387	1.0000
Aug	Sep	−12.6111	−8.1503	−3.6896	0.0000
Aug	Oct	−10.2207	−5.7600	−1.2993	0.0015
Aug	Nov	−5.9487	−1.4879	2.9728	0.9951
Aug	Dec	2.0596	6.5203	10.9811	0.0001
Sep	Oct	−2.0704	2.3903	6.8511	0.8441
Sep	Nov	2.2017	6.6624	11.1231	0.0001
Sep	Dec	10.2100	14.6707	19.1314	0.0001
Oct	Nov	−0.1887	4.2721	8.7328	0.0755
Oct	Dec	7.8196	12.2803	16.7411	0.0001
Nov	Dec	3.5475	8.0083	12.4690	0.0001

## References

[1] Oyedepo S.O., Adekeye T., Leramo R.O., Kilanko O., Babalola O.P., Balogun A.O., Akhibi M.O. (2016). Assessment of energy saving potentials in covenant university, Nigeria. Energy Eng.: J. Assoc. Energy Eng..

[2] A.A. Ogundipe, O. Akinyemi, O.M. Ogundipe, Electricity consumption and economic development in Nigeria, Int. J. Energy Econ. Policy, vol. 6(1), pp. 134–143.

[3] Oyedepo S.O., Adaramola M.S., Odunfa M.K., Aremu O.T. (2015). Analysis of Energy Utilization in Selected Industries in Southwestern Nigeria. Energy Eng.: J. Assoc. Energy Eng..

[4] Oyedepo S.O. (2014). Towards achieving energy for sustainable development in Nigeria. Renew. Sustain. Energy Rev..

[5] Oyedepo S.O. (2012). Efficient energy utilization as a tool for sustainable development in Nigeria. Int. J. Energy Environ. Eng..

[6] Oyedepo S.O. (2012). Energy and sustainable development in Nigeria: the way forward. Energy, Sustain. Soc..

[7] Oyedepo S.O. (2012). On energy for sustainable development in Nigeria. Renew. Sustain. Energy Rev..

[8] A.A. Atayero, V. Ademu-Eteh, S.I. Popoola, T.O. Takpor, J.A. Badejo, Occupancy controlled lighting system for smart buildings, in: Proceedings of The World Congress on Engineering and Computer Science 2017, San Francisco, USA, Lect. Notes Eng. Comput. Sci., 25–27 October, 2017, pp. 707–710.

[9] S.I. Popoola, O.A. Popoola, A.I. Oluwaranti, J.A. Badejo, A.A. Atayero, A framework for electronic toll collection in smart and connected communities, in: Proceedings of The World Congress on Engineering and Computer Science 2017, Lect. Notes Eng. Comput. Sci., San Francisco, USA, 25–27 October, 2017, pp. 723–726.

[10] A.U. Adoghe, I.O. Oyinlola, S.I. Popoola, A.A. Atayero, Free energy generation using neodymium magnets: an off-grid sustainable energy solution for Sub-Saharan Africa, in: Proceedings of the World Congress on Engineering 2017, Lect. Notes Eng. Comput. Sci., London, U.K., 5–7 July, 2017, pp. 277–282.

[11] V.O. Matthews, Q. Osuoyah, S.I. Popoola, E. Adetiba, A.A. Atayero, C-BRIG: a network architecture for real-time information exchange in smart and connected campuses, in: Proceedings of The World Congress on Engineering 2017, Lect. Notes Eng. Comput. Sci., London, U.K., 5–7 July, 2017, pp. 398–401.

[12] S.O. Oyedepo, T. Adekeye, R. Leramo, O. Kilanko, P. Babalola, A study on energy demand and consumption in Covenant University Ota, Nigeria, in: Proceedings of the International Conference on African Development Issues (CU-ICADI), Ota, Nigeria, 11–13th May, 2016, pp. 203–211.

